# Statistical evidence that honeybees competitively reduced wild bee abundance in the Munich Botanic Garden in 2020 compared to 2019

**DOI:** 10.1007/s00442-022-05113-y

**Published:** 2022-01-25

**Authors:** Susanne S. Renner, A. Fleischmann

**Affiliations:** Ludwig-Maximilians-Universitat Munchen, Munich, Germany

**Keywords:** Exploitative competition, Honeybees, Wild bees, Food competition, Competitive displacement, Urban bee keeping

## Abstract

In a commentary on our paper (Renner et al., Oecologia 195:825–831, 2021), Harder and Miksha lay out why they think that our finding of higher honeybee abundances reducing wild bee abundances in an urban botanical garden is not statistically supported. Here, we explain the statistical test provided in our paper, which took advantage of a natural experiment offered by 2019 being a poorer year for bee keeping than 2020.

## Introduction

Despite much concern over the possibility that urban beekeeping may lead to unsustainably high bee abundances, resulting in foraging honeybees displacing wild bees from flowers (Weissman et al. 2021 for a recent review), statistically supported findings have been lacking. To study honeybee and non-honeybee densities at flowers in an urban setting—regardless of the flowers’ pollination syndromes—we offered bachelor thesis projects in the botanical garden of Munich, which has been in its current location since 1914 and offers a stable set of ornamental and native species in its display gardens and meadows. Each of the bachelor projects was limited to about 6 weeks of field work, during which the students counted bee visits to flowers or inflorescences for a total of about 9 h per species, with the number of flowers chosen so that all bees could be seen and counted with precision. Observations were only made during dry, sunny or at most slightly overcast days. Our initial goal was to find out the relative abundances of both groups of bees at flowers, and the working hypothesis was that there would be strong niche separation because of flowers’ different suitability for honeybees versus wild bees in terms of flower sizes, flower morphology, and flower numbers. Results in 2019, however, showed surprisingly little niche separation. Instead, honeybees visited all types of flowers (Renner et al. 2021: Table 1). To test this unexpected result, the observations were repeated in 2020. We expected there would be natural fluctuation in the abundances of wild bees as well as honeybees in 2020 compared to 2019, and that relative abundances at flowers would, therefore, be different, with some flowers receiving more wild bee visits, others more honeybee visits.

However, this expectation again was not met. Instead, abundances of the two types of bees remained unchanged in April 2020 compared to April 2019, but from May to July 2020, honeybee abundances consistently increased compared to 2019, mostly due to natural reproduction (swarming of bees). We carried out a chi-square test of independence that compared the number of observed shifts in visitor spectra in April with those in May–July (Renner et al. 2021: p. 828) to test the expectation that under food competition, increased honeybee densities would shift the relative proportions of wild bees and honeybees. The results showed reduced numbers of wild bee visits in nine of 20 May/June/July-flowering species, while visitor spectra did not change in the ten April-flowering species (as we reported, Renner et al. 2021: Table 1, this shows 30 observations, because *Nepeta mussinii* was observed in April and May; *χ*^2^ = 6.43, df = 1, *P* = 0.05 (As usual, df = (r − 1)(c − 1), where r is the number of rows and c is the number of columns.) All observed shifts in visitor spectra were in the direction of increased honeybee numbers.

## Conclusion

We are grateful to Harder and Miksha (2021) for their critical comments, but stand by our interpretation that our study demonstrates the depressing effects of increased honeybee densities on the simultaneous proportions of wild bees at flowers of the same species. However, as we pointed out, we lack data on the fitness effects of this observation. It is plausible that in the summer of 2020, wild bees had to travel further and/or use less profitable flowers compared to 2019, but to determine whether this had non-trivial effects on their fitness would require competitive exclusion experiments combined with longer term studies of wild bee populations. To our knowledge, no such study has been carried out. That the visitor shifts observed in 2020 might instead have been due to lower abundances of wild bee species, or to higher or lower flower densities, seems implausible given the complete consistency of the direction of shifts (from wild bees to honeybees) throughout all 3 months with higher honeybee densities, and the stable supply of nectar and pollen in the botanical garden, where dense plantings of ornamentals are maintained year-round in fertilized soil that is always kept watered.

## Data Availability

The data analyzed for this comment were presented in Table 1 of Renner et al. (2021).
